# Biodegradable sustained-release microneedle patch loaded with clindamycin hydrochloride: a breakthrough in acne management

**DOI:** 10.3389/fphar.2025.1575635

**Published:** 2025-05-30

**Authors:** Haomei Fan, Ruohan Liao, Yiling Yang, Yan Xing, Chengdong Zhang, Xuwei Luo, Chao Pu, Liling Wu, Xingping Li, Juhua Zhao, Dongqin Xiao

**Affiliations:** ^1^ Department of Dermatology, Research Institute of Tissue Engineering and Stem Cells, The Second Clinical College of North Sichuan Medical College, Nanchong, China; ^2^ School of Clinical Medicine, Southwest Medical University, Luzhou, China; ^3^ Department of Dermatology, Mianyang Maternal and Child Healthcare Hospital (Mianyang Children’s Hospital), Mianyang, China; ^4^ Department of Orthopaedics, Chengfei Hospital, Chengdu, China

**Keywords:** acne vulgaris, swellable microneedles, GelMA hydrogel, clindamycin hydrochloride, minimally invasive drug delivery system

## Abstract

**Background:**

Clindamycin hydrochloride, a first-line antibiotic for acne treatment, faces challenges with poor skin penetration due to its hydrophilicity and the barrier posed by the stratum corneum. To address this limitation, we developed gelatin-methacryloyl (GelMA) hydrogel-based biodegradable microneedles (GM-Clin-MN) for sustained intradermal drug delivery, thereby enhancing therapeutic efficacy.

**Methods:**

The microneedle patches loaded with 1 wt% clindamycin hydrochloride were fabricated using PDMS molds and characterized through scanning electron microscopy (SEM), Fourier-transform infrared spectroscopy (FTIR), and fluorescence microscopy. Drug loading and release were assessed using UV-Vis spectroscopy at 520 nm, while mechanical strength was evaluated with a universal testing machine. Skin penetration was tested on *ex vivo* rat abdominal skin. Biosafety was determined through human skin fibroblast (HSF) cytotoxicity and hen’s egg test-chorioallantoic membrane (HET-CAM) irritation tests. Antibacterial efficacy against Cutibacterium acnes (*C. acnes*) was measured via colony counting. *In vivo* acne treatment of the microneedles was evaluated in a rat acne model. Gross morphological changes, histological sections, and immunohistochemical staining were used to evaluate the efficacy and potential mechanisms of acne treatment.

**Results:**

Clindamycin hydrochloride-loaded GelMA microneedles (GM-Clin-MN) achieved a drug loading of 0.49 ± 0.025 μg/needle, exhibiting rapid release on Day 1 (54.8% ± 2.1%) and sustained release by Day 10 (72.1% ± 1.5%). The microneedles penetrated the skin to a depth of 658 ± 66 μm, swelled by 185.4% ± 12.1%, and completely dissolved within 10 min. GM-Clin-MN displayed no cytotoxicity or skin irritation and effectively inhibited the growth of *C. acnes* (bacterial inhibition rate of 100%). *In vivo* studies revealed that acne-related inflammation was effectively suppressed with potential anti-scarring properties, characterized by reduced pro-inflammatory IL-1β levels, increased anti-inflammatory IL-10 expression, and diminished MMP-2 activity — a key enzyme in collagen overproduction during scarring.

**Conclusion:**

GM-Clin-MN enables sustained, minimally invasive clindamycin delivery through the stratum corneum, offering a dual-action therapeutic strategy that combines potent antibacterial activity with anti-inflammatory modulation for acne management.

## 1 Introduction

Acne vulgaris is a common chronic inflammatory skin disease that affects individuals worldwide, especially adolescents. Severe to moderate acne can lead to skin pigmentation and even leave permanent scars, causing negative psychological and social impacts on patients, such as stress, embarrassment, and a blow to self-esteem (Ali et al., 2024). The treatment of acne typically involves oral medications, topical treatments, and physical therapies (Layton and Ravenscroft, 2023). Topical therapy is the first-line treatment for mild to moderate acne, including agents such as retinoids, benzoyl peroxide, vitamin A derivatives, and antibiotics. The use of topical tretinoin cream can easily lead to redness and swelling at the application site, as well as the dilation of blood vessels. In some cases, it may also be accompanied by a certain degree of peeling or flaking skin. When severe, it can result in skin sensitivity in the treated area and a weakening of the skin’s barrier function (Narsa et al., 2024). Clindamycin topical formulations (solutions, creams, and gels) have become the predominant therapeutic modality for acne vulgaris management, owing to their patient compliance advantages, favorable safety profile, and minimized first-pass metabolism effects (CDA-AMC, 2025). As the predominant driver of inflammatory acne, *Cutibacterium acnes (Cutibacterium acnes)* demonstrates heightened susceptibility to clindamycin, exhibiting greater potency compared to erythromycin (Niedźwiedzka et al., 2024), while concurrently suppressing key inflammatory mediators including IL-8 and TNF-α through ribosomal targeting and immune modulation (Armillei et al., 2024). This dual antimicrobial/anti-inflammatory profile positions clindamycin as a cornerstone therapy. Nevertheless, clindamycin exhibits three key limitations in clinical practice: its strong hydrophilicity results in low transdermal efficiency, hindering stratum corneum penetration and effective dermal drug accumulation (Elhabal et al., 2024); prolonged use induces *C. acnes* resistance (Ghannoum et al., 2025); and local irritation restricts long-term application (Feldman et al., 2025). Therefore, developing a transdermal delivery system that enhances clindamycin’s therapeutic efficacy while minimizing local irritation is critically needed.

Microneedle (MN) technology employs micrometer-sized needles (50–1,000 μm) to penetrate minimally invasively the stratum corneum, enabling targeted drug delivery to the dermis. Since the development of the first solid microneedle device in 1971, MN systems have evolved into five functional categories: solid, coated, hollow, dissolving, and swellable microneedles (Nguyen and Banga, 2024; Wang et al., 2024). Solid microneedles create transient microchannels for passive drug diffusion after insertion and removal, while coated variants deliver drugs pre-deposited on their surfaces. Hollow microneedles utilize pressurized flow through their hollow cores for active drug release, and dissolving types degrade within the skin to release encapsulated payloads. In contrast, swellable microneedles—composed of crosslinked hydrogel polymers—absorb interstitial fluid to swell and controllably release drugs through matrix degradation (Chauhan et al., 2024).

Traditional microneedle materials (e.g., silicon or metals) face limitations including poor drug release modulation, complex fabrication processes, cytotoxicity risks, and skin irritation (Kumar et al., 2023). These challenges are particularly critical for therapies like clindamycin-mediated acne treatment, which demand sustained antibiotic exposure at pilosebaceous units to maintain therapeutic efficacy while minimizing local irritation. Swellable microneedles address these needs through their unique swelling-triggered release mechanism. Their crosslinked hydrogel structure provides high biocompatibility, tunable drug-loading capacity, and programmable release kinetics (Omidian and Dey Chowdhury, 2025; Mohite et al., 2024; Jin et al., 2023), directly overcoming the dual limitations of conventional topical systems-suboptimal transdermal permeation and imprecise dosing control. Beyond pharmacological advantages, swellable microneedles enhance acne patient compliance by eliminating injection-associated pain (as they avoid contact with nerve endings and blood vessels) and reducing infection risks (Rai et al., 2023). Their versatility has expanded applications to immunotherapy, diagnostic monitoring, and cosmetic enhancements (Omidian and Dey Chowdhury, 2025), positioning them as a transformative platform in minimally invasive transdermal delivery.

**FIGURE 1:**
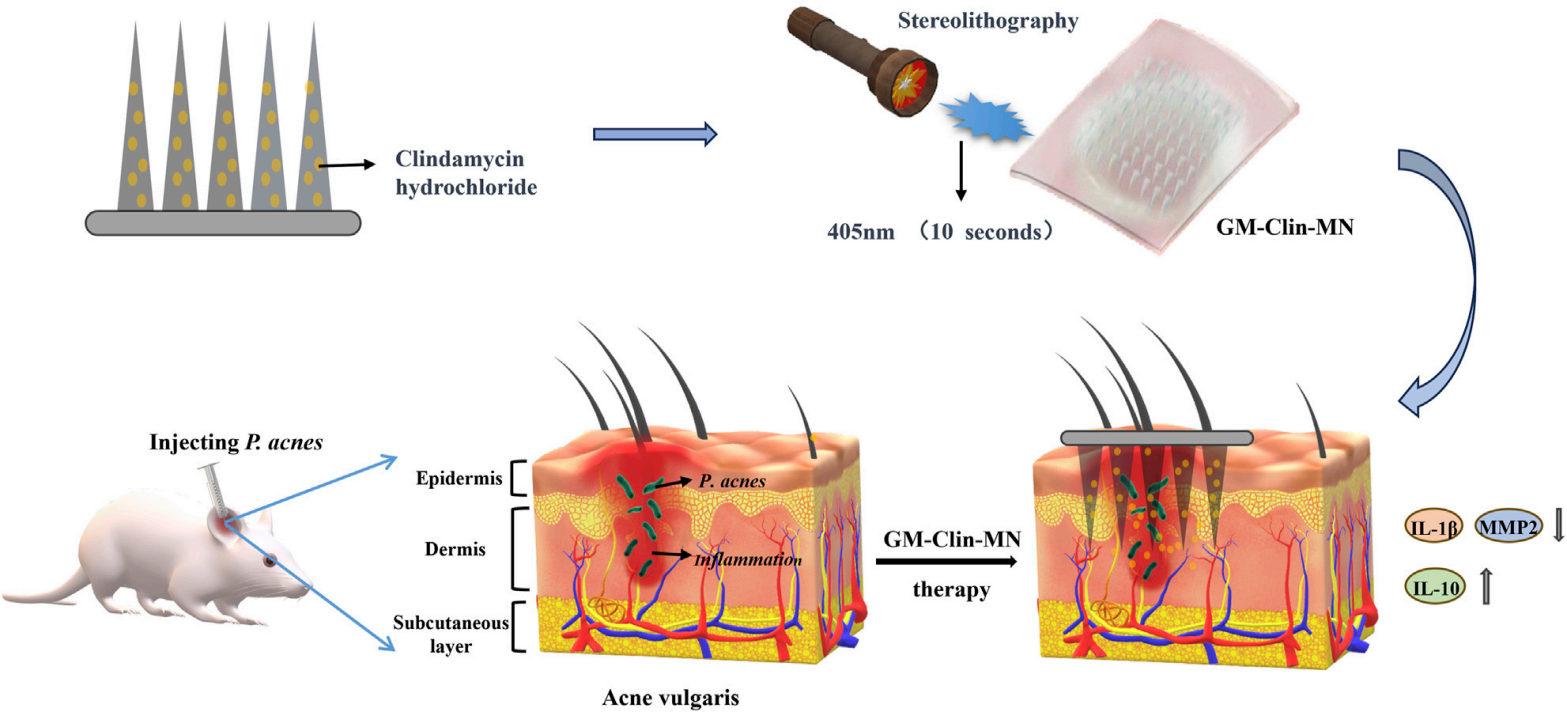
Schematic illustration of the formation and mechanism of GM-Clin-MN patch for acne vulgaris treatment.

Therefore, to enhance the utilization rate of clindamycin and improve the treatment efficacy for acne, this study aims to develop a gelatin-methacryloyl (GelMA)-based swellable microneedle system for enhanced transdermal delivery of clindamycin, addressing the critical limitations of current acne therapies through controlled drug release and improved skin targeting (as shown in Figure 1). GelMA is synthesized by introducing methacryloyl groups onto the amino side chains of gelatin, which endows it with unique photo-crosslinking properties, as well as controllable mechanical, degradation, and biocompatibility characteristics (Pramanik et al., 2024; Wang et al., 2025; Das et al., 2024). Consequently, this study employed GelMA to engineer clindamycin-loaded microneedles, which formed a cohesive microneedle patch following blue light irradiation. The patch demonstrated effective penetration into the dermal layer of rat skin. As the GelMA needle tips underwent swelling and gradual degradation *in vivo*, the encapsulated drug achieved sustained release within the dermis, thereby exerting anti-inflammatory effects and mitigating acne lesions.

## 2 Materials and methods

### 2.1 Materials

All solvents used for isolation and purification were of ACS reagent grade (Sigma-Aldrich Chemical Co., Germany). Porcine gelatin (Type A, 300 bloom from porcine skin), polyvinyl alcohol (PVA) and methacrylic anhydride were purchased from Sigma-Aldrich (MO, United States). Photoinitiator LAP (Lithium Phenyl (2,4,6-trimethylbenzoyl) phosphinate) was purchased from EFL Company (Engineering for Life Co., Ltd., Suzhou, China). Polydimethylsiloxane (PDMS, Model: A73) mold was sourced from Taizhou Microchip Pharmaceutical Technology Co., Ltd. (Taizhou, China). Human skin fibroblast (HSF) and clindamycin hydrochloride were obtained from Shanghai Hongshun Biotechnology Corporation (Shanghai, China). Fertilized chicken eggs were purchased from Sichuan Yixinhe Poultry Breeding Corporation (Chengdu, China). The *C. acnes* (ATCC 6919) strain was obtained from the Guangdong Provincial Center for Microbial Culture Collection (Guangdong, China). Deionized water was used throughout the experimental process. All chemicals were of analytical grade.

### 2.2 Synthesis of GelMA

In this experiment, 20 g of porcine gelatin was placed in a 500 mL beaker, followed by the addition of 200 mL of phosphate-buffered saline (PBS). The reaction mixture was continuously agitated at 40°C with a magnetic stirrer operating at 400 rpm for 12 h. Subsequently, the temperature was raised to 45°C, and 16 mL of methacrylic anhydride (MAA) was added dropwise in the dark, with stirring for an additional 2 h. After centrifugation at 9,000 rpm for 5 min to collect the supernatant, the solution was dialyzed for 7 days, with daily water changes. Following dialysis, the solution was frozen at −80°C overnight and then freeze-dried to obtain GelMA. For analysis, 10 mg each of gelatin and GelMA were dissolved in 1 mL of deuterated water at 40°C and subjected to proton spectroscopy in a Nuclear Magnetic Resonance (NMR, Bruker-400, Bruker Dimension ICON, Germany) tube. Additionally, 100 mg of GelMA was dissolved in 1 mL of deionized water at 40°C, mixed with 2 mg of photoinitiator LAP and 1 mg of rhodamine 6G, and examined for morphological changes and crosslinking after exposing 500 μL of the mixture to blue light for 10 s, with another 500 μL serving as an untreated control.

### 2.3 Preparation of microneedle patches

Preparation of the base layer stock solution: 20 g of PVA was weighed and added to 100 mL of deionized water. The mixture was stirred at 90°C until complete dissolution, followed by standing overnight to eliminate residual bubbles, yielding a 20% (W/V) PVA solution.

Preparation of tip stock solutions: For the blank microneedle patch (GM-MN), lyophilized GelMA (100 mg) was dissolved in 1 mL deionized water at 40°C, followed by addition of 2 mg LAP. The mixture was stirred until homogeneous and stood overnight for bubble removal. For the clindamycin hydrochloride-loaded microneedle patch (GM-Clin-MN), 100 mg lyophilized GelMA dissolved in 1 mL deionized water at 40°C was blended with 2 mg LAP and 10 mg clindamycin hydrochloride. The solution was stirred thoroughly to uniformity and left undisturbed overnight to eliminate bubbles.

The custom-designed PDMS mold (Model: A73; Taizhou Microchip Pharmaceutical Technology Co., Ltd., Taizhou, China) contains microneedle cavities with the following specifications:array configuration: 20 × 20 needles (400 needles per patch); needle geometry: square pyramidal (quadrangular frustum); vertical height: 1,000 μm; base dimensions: 500 μm × 500 μm; inter-needle spacing: 800 μm (center-to-center); patch area: 25 mm × 25 mm; mold cavity depth: 2 mm. The microneedle patches were fabricated as follows: First, 200 μL of the tip stock solution was dispensed into a PDMS mold. To ensure complete filling of the microneedle cavities, the mold was subjected to vacuum drying at 40°C (−0.08 MPa, DZF-6020 chamber, Shanghai Jingqi Instrument Co., Ltd., China) for 2–3 cycles, effectively removing air bubbles and driving the solution into the needle structures. Excess solution containing residual bubbles on the mold surface was then aspirated, followed by a 2 h oven-drying step at 30°C to concentrate the matrix. To reinforce structural integrity, an additional 100 μL of stock solution was dispensed and further concentrated under identical drying conditions for another 2 h. Photocrosslinking was subsequently performed by irradiating the filled mold with 405 nm UV light for 10 s. A backing layer was formed by applying 200 μL of 20% (w/v) PVA solution onto the cured microneedle array. The composite structure was then dried at 30°C for 12 h to achieve mechanical stability, after which the fully cured microneedle patch was gently demolded from the PDMS template.

### 2.4 Characterization

The overall image of the microneedles was captured using a camera, while the microscopic morphology of the microneedles was examined through a scanning electron microscope (SEM, FEI Quanta 450 FEG) and fluorescence microscopy. ImageJ software was employed for the analysis of the microneedle dimensions. The composition of the GelMA was analyzed through NMR spectroscopy spectra (Bruker-400) using D_2_O as the solvent. Fourier-transform infrared spectroscopy (FT-IR, Nicolet IS5, Thermo Fisher Scientific, Waltham) was used to evaluate the chemical composition of the samples. Mechanical property testing was conducted using an electronic universal testing machine (C45.105, MTS, China), with a probe-to-needle distance set at 2.0 mm and a probe downward speed of 1.0 mm/min. The mechanical data were recorded by the sensor at a frequency of 200 times per second. UV-visible spectrophotometry (UV-2600, Shimadzu Corporation, Japan) was used to quantify the drug loading capacity.

### 2.5 *In vitro* release assay

For the *in vitro* release, the clindamycin-loaded microneedle patch was sealed in a dialysis bag (MWCO 8–10 kDa) and immersed in 10 mL of pH 7.4 phosphate-buffered saline (37°C, 200 rpm) (Figure 3k). At predetermined intervals (1 h–10 days), 3 mL of release medium was collected and replaced with fresh buffer. The collected samples were processed identically to the standard curve method, and the cumulative release rate was calculated based on absorbance measurements. Specifically, the quantification of clindamycin hydrochloride was performed using UV-Vis spectroscopy (Shimadzu UV-2700i, Japan) at 520 nm, based on the oxidation of its methylthio group by potassium iodate under acidic conditions (El-Yazbi and Blaih, 1993). For the standard curve, a 2 mg/mL stock solution was prepared by dissolving 100 mg of clindamycin hydrochloride reference standard in deionized water (50 mL volumetric flask). Aliquots of 1–5 mL of the stock solution were transferred to 25 mL volumetric flasks, each treated with 10 mL of 1% (g/mL) cyclohexane, 3 mL of 30% sulfuric acid, and 4 mL of potassium iodate solution, followed by heating in a 60°C water bath for 45 min. After cooling, the cyclohexane layer was extracted three times with fresh solvent, and the combined extracts were transferred to a 25 mL volumetric flask, diluted to the mark, and thoroughly mixed. Absorbance at 520 nm was measured in triplicate for each sample. A linear regression equation was established as C = 1.034 A + 0.0166 (R^2^ = 0.999), where C represents concentration (mg/mL) and A denotes absorbance, demonstrating a linear range of 0.1–0.5 mg/mL.

To determine drug loading, the microneedle patch was incubated in 3 mL of GelMA lysate at 37°C for 60 min, centrifuged (12,000 rpm) to collect the supernatant, and analyzed using the same UV spectrophotometric procedure to calculate the drug loading capacity.

### 2.6 *In vitro* skin penetration assay

To assess the skin penetration ability of the microneedles *in vitro*, the rhodamine 6G-loaded microneedle patch was applied to the rat abdominal skin without hair, which was mounted onto a foam board. After exerting continuous pressure for 1 min, the treated area was gently cleansed with a moist cotton swab to ensure the removal of excess dye. Skin surface images were captured, and insertion ratios were calculated. Tissue sections underwent H&E staining to observe the depth of penetration by the microneedles. All experiments were performed in triplicate.

### 2.7 Swelling and separation performance of needle tips

The GM-Clin-MN patch was placed onto the abdominal skin of SD rats. Detachment of the microneedle tips from the substrate backing layers was observed at 10 min during preliminary experiments. Therefore, the observation time points were set at the first minute of insertion, the partial detachment time (5 min), and the complete detachment time (10 min). After specific time intervals (e.g., 1 min, 5 min, 10 min), the patch was removed, and the morphological changes in the needle tips were captured under a microscope. To determine the swelling ratio of GM-Clin-MN micro-needles, the GM-Clin-MN microneedles were placed in 5 mL of PBS solution at 37°C in the past. The microneedles were allowed to reach equilibrium swelling. The post-swelling mass (W_1_) and the initial mass (W_2_) of the microneedles were measured. The swelling ratio (S) was calculated using the following formula:

$$S=W_{1}/W_{2}.$$

where W1 represented the mass of the microneedles after swelling, and W2 represented the mass of the microneedles before swelling.

### 2.8 Cytotoxicity evaluation

GM-MN and GM-Clin-MN were cut into 1 cm diameter circular discs and sterilized with 12 h of UV exposure. These micro-needle patches were placed in a 24-well plate with 6 wells per group. Each well was seeded with 100 µL of P5 passage HSF suspension at a density of 2 × 10^4^ cells/well. The plate was then incubated at 37°C and 5% CO_2_ for 1 h. Afterward, 400 µL of DMEM culture medium (containing 1% penicillin-streptomycin and 10% fetal bovine serum) was added to each well, and incubation continued for 24 h. After removing the supernatant, cells were washed three times with PBS. Subsequently, 200 µL of Cell Counting Kit-8 (CCK-8, Dojindo Molecular Technologies, Japan) detection solution and 1.8 mL of DMEM culture medium were added to each well. The plate was placed in a cell culture incubator at 37°C and 5% CO_2_ for 1 h. After incubation, 100 µL of the solution was collected and measured at 450 nm using an microplate reader. The proliferation of HSF was also measured using the same method on day 4 and 7.

### 2.9 Hen’s egg test-chorioallantoic membrane (HET-CAM) experiment

The experiment was divided into four groups: the negative control group, positive control group, GM-MN group, and GM-Clin-MN group, with six chicken embryos per group. Seven-day-old fertilized chicken embryos were selected, and a circular window with a diameter of 1 cm was created on the air chamber end to expose the chorioallantoic membrane (CAM) blood vessels. The endothelium was removed, and the CAM blood vessels were exposed using forceps, ensuring that the blood vessels were bare. A sterilized glass slide was used to add 100 μL of the test solution (10% GelMA, 1% Clin, 0.2% LAP) with a pipette. Then, the solution was exposed to blue light for 10 s, forming a solid disk. In a clean laminar flow hood, 100 μL of PBS solution was added to the CAM as the negative control group, and 100 μL of 10% NaOH solution was added to the CAM as the positive control group. The sample was placed in the central position of the CAM in the experimental group. Prior to the experiment and at 30 s, 120 s, and 300 s during the experiment, photographs of the CAM were captured using a stereomicroscope. Observations were made to check for signs of bleeding, clotting, or blood vessel dissolution. Endpoints of hemorrhage, vasoconstriction, and coagulation of each group were monitored in order to calculate the irritation score (IS), as following Equation 1:

$$\text{IS}=\left[5\times \left(301\;-\text{ hemorrhage time}\right)/300\right]+\left[7\times \left(301\;-\text{ vasoconstriction time}\right)/300\right]+\left[9\times \left(301\;-\text{ coagulation time}\right)/300\right]$$ (1)

The results were expressed as the mean of IS and relative standard deviation in percentage (RSD%) and were classified by the mean as non-irritant [0.0–0.9], slightly irritant [1.0–4.9], moderately irritant [5.0–8.9], and extremely irritant [9.0–21.0].

### 2.10 *In vitro* antimicrobial experiment

The prepared GM-MN and GM-Clin-MN were cut into circular disks with a 1 cm diameter and sterilized by exposing them to UV light for 12 h. These microneedle patches were then placed in a 24-well plate, with 6 parallel wells per group. In each well, 500 μL of *C. acnes* bacterial suspension (concentration of 1 × 10^6^ CFU/mL) was added to promote bacterial growth on the microneedle patches. A control group was prepared by adding 500 μL of PBS culture medium to the corresponding wells. Place the Petri dish in an anaerobic bag and incubate in a constant temperature shaking incubator at 37°C for 6 h to facilitate bacterial growth on the microneedle patch. Afterwards, 50 μL of the co-cultured bacterial suspension was taken and inoculated on Brain Heart Infusion (BHI) solid culture medium. According to the manufacturer’s protocol, *C. acnes* requires 48-h anaerobic incubation at 37°C. To exclude potential interference from environmental factors that may compromise bacterial growth, the cultivation duration was extended to 72 h in this study. The plates were then placed in anaerobic bags and incubated in a 37°C constant temperature incubator for 72 h. This experiment was repeated three times. Following the incubation period, results were captured and recorded by taking photographs.

### 2.11 *In vivo* anti-acne experiment

*In vivo* anti-acne experiments were conducted using 5-week-old male SD rats purchased from the Experimental Animal Center of North Sichuan Medical College. All animal procedures were performed in accordance with the Chinese national standard “Guidelines for the Ethical Review of Animal Welfare in Experimental Studies” (GB/T 35892-2018) and were approved by the Animal Ethics Committee of North Sichuan Medical College (approval no. NSMC2024020). Briefly, a 2% coal tar solution was applied to the inner surface of the right ear of SD rats once daily. The following day, *C. acnes* bacterial solution (1 × 10^6^ CFU/mL) was injected subcutaneously into the right earlobe, approximately 100 μL per injection, every other day for a total of 14 days, to establish the acne model. After modeling, the rats were randomly divided into four groups. The groups consisted of a negative control (PBS application) group, a blank microneedle (GM-MN) group, a clindamycin hydrochloride gel (Clin-Gel) group, and a clindamycin hydrochloride-loaded microneedle (GM-Clin-MN) group, with 10 rats in each group. In the negative control (PBS application) group, sterile PBS solution was applied to the lesion area with a cotton swab, once in the morning and once in the evening. In the Clin-Gel group, clindamycin gel was applied to the lesion area with a cotton swab, once in the morning and once in the evening. In the GM-MN and GM-Clin-MN groups, the microneedle patch was cut into a square array of 15 × 15 needle tips, pressed and attached to the acne lesions on the rat’s ear. The microneedle patch was administered every other day. Each group was treated for a total of 9 days. On the 0, 3, 6, and 9 days of treatment, the ear thickness of the experimental rats was measured using a vernier caliper and recorded. Photographs were taken on the 0 and 9 days of treatment.

After treatment, ear lesions were excised from three independent biological replicates per group (n = 3) and fixed with 4% paraformaldehyde (PFA) for histological analysis. Tissue specimens were embedded in paraffin and cut into 5 μm sections. These sections were mounted on glass slides and stained with hematoxylin and eosin (H&E) for general histological examination. Histopathological scoring was performed by comparing lesional tissues with normal rat auricular tissue using the following criteria:

Individual parameter grading: 0: No abnormalities, 1: Mild, 2: Moderate, 3: Severe.

Cumulative score classification: 0: − (Normal); 1–5: + (Mild); 6-10: ++ (Moderate); 11–15: +++ (Severe).

The HE-stained sections were independently assessed by ≥2 board-certified pathologists in a blinded manner (with group allocation concealed) using a standardized scoring system. The mean scores were calculated based on the recorded values of all evaluated parameters.

To further assess the inflammatory status at the site of *C. acnes* infection, immunohistochemical staining was performed using the following primary antibodies: anti-interleukin-1β (IL-1β) (ab9722, Abcam), anti-(matrix metalloproteinase-2 (MMP-2) (DF6998, Affinity), anti-interleukin-10(IL-10) (ab97779, Abcam), and Goat anti-Rabbit IgG (111-035-045, Jackson) as the secondary antibody. Three inflammatory factors, IL-1β, MMP2, and IL-10, were targeted for detection. For immunohistochemical analysis, paraffin-embedded sections (5 μm) were first blocked with goat serum and then incubated overnight at 4°C with the aforementioned primary antibodies. Afterward, the sections were incubated with the secondary antibody, Goat anti-Rabbit IgG, and the targets were visualized using 3,3′-diaminobenzidine (DAB) staining. Following staining, the sections were counterstained with hematoxylin, dehydrated through a series of alcohol gradients, cleared in xylene, and mounted with a resin mounting medium. The sections were examined under a light microscope, with positive signals for IL-1β, MMP-2, and IL-10 proteins appearing as brown particles. The integral optical density (IOD) values of IL-10, IL-β, and MMP protein expression were quantified using ImageJ software.

### 2.12 Statistical analysis

The experimental data obtained were analyzed using SPSS 23.0 software. The experimental data are presented as mean ± standard deviation (Mean ± S.D.). For comparisons between two groups, a t-test was employed. For comparisons among multiple groups, one-way analysis of variance (ANOVA) was used. Post-hoc multiple comparisons were conducted using the Tukey method. A significance level of *p < 0.05* was considered statistically difference.

## 3 Results

### 3.1 Synthesis and characterization of GelMA

**FIGURE 2:**
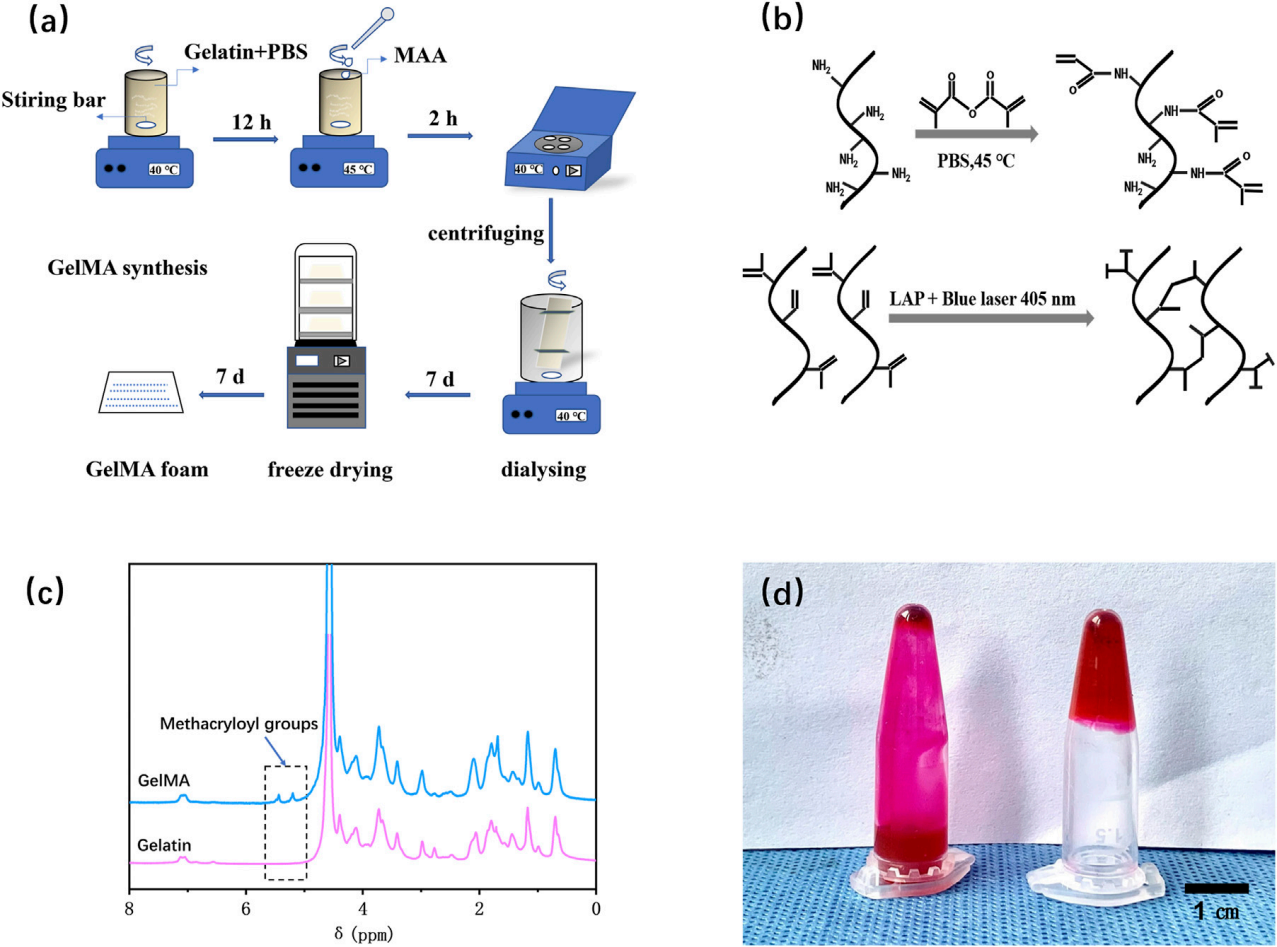
Fabrication and characterization of GelMA. **(a)** Schematic illustration of the preparation procedures for GelMA. **(b)** The chemical synthesis principle of GelMA. **(c)** NMR spectra of gelatin and GelMA. **(d)** Verification of the photo-crosslinking characteristics of GelMA.

Figures 2a,b depict the synthesis steps and principle of GelMA. Figure 2c displayed the NMR spectrum of gelatin and GelMA, where the characteristic peak of GelMA (5.5 ppm) was indicated by the dashed box. The substantial substitution of amino groups with methacryloyl groups from methacrylic anhydride indicated the successful preparation of GelMA. According to the literature (Li et al., 2016), the calculation formula for the amino substitution degree (DM) on the gelatin side chains by methacrylic anhydride was DM=(I_5.5_ppm/I_0.8_ppm) ×100%. The calculated result was 72.85%. Figure 2d validated the photo-crosslinking properties of GelMA. As shown, GelMA labeled with rhodamine 6G dye remained in a liquid state in solution without blue light exposure but transformed into a gel state even when inverted after blue light irradiation. This indicates that the prepared GelMA possesses photo-crosslinking characteristics.

### 3.2 Synthesis and characterization of GM-clin-MN

**FIGURE 3:**
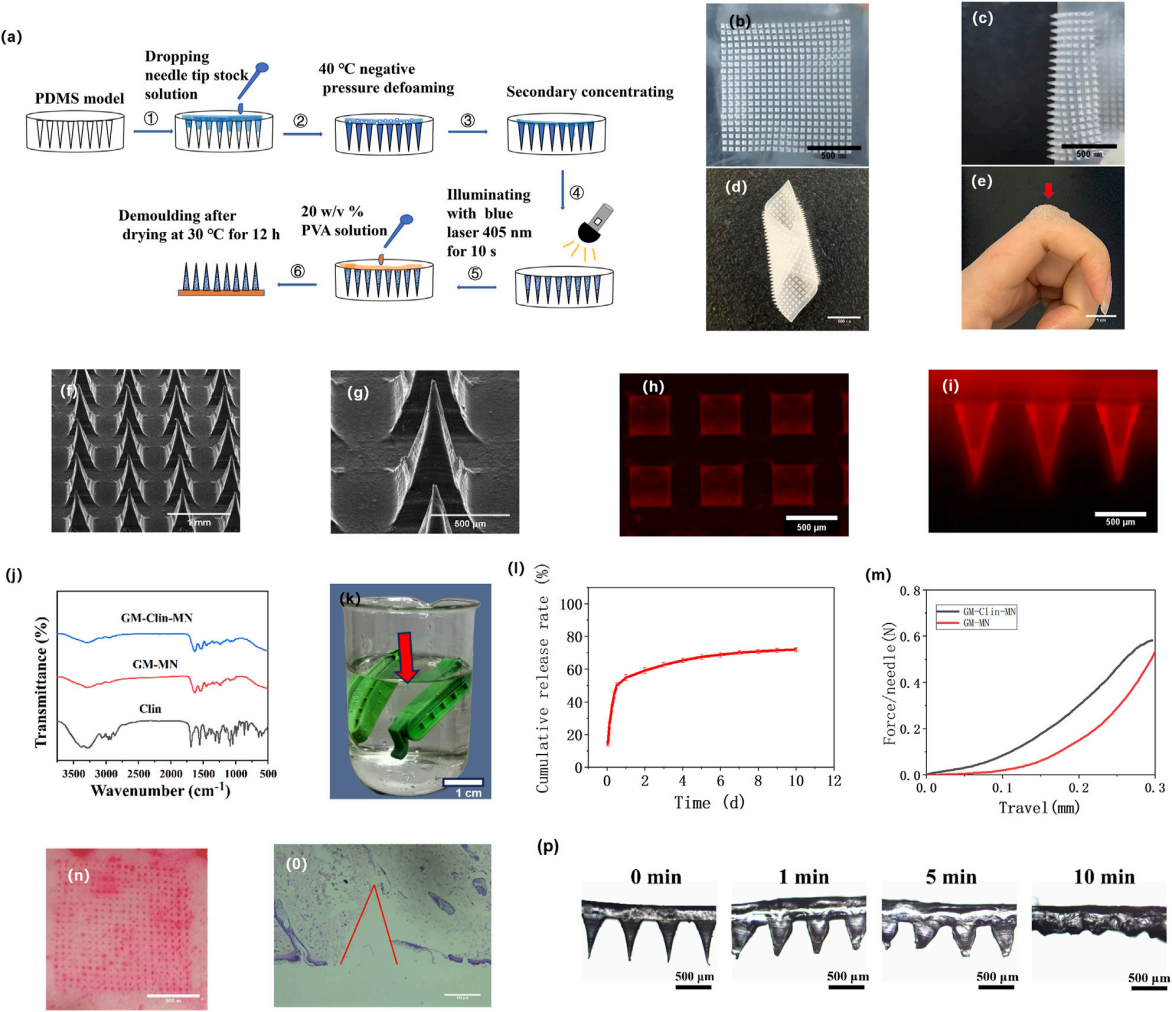
Fabrication and characterization of GM-Clin-MN. **(a)** Schematic illustration of the preparation procedures for GM-Clin-MN. **(b–e)** Digital photograph of the GM-Clin-MN patch **(f,g)** SEM images of GM-Clin-MN. **(h,i)** Fluorescence microscopy images showing rhodamine-labeled MN. **(j)** FT-IR spectra of GM-Clin-MN, GM-MN and Clin. **(k)** Schematic diagram of GM-Clin-MN (red arrow) enclosed in a dialysis bag submerged in phosphate buffer solution **(l)** *In vitro* release curve of clindamycin hydrochloride. **(m)** Mechanical strength-displacement curve of the microneedles. **(n)** Image of rat abdomen skin after piercing with microneedles labeled with Rhodamine 6G. **(o)** Histopathological image of skin after microneedle piercing, stained with hematoxylin and eosin (H&E). **(p)** Optical microscopy images of microneedle tip morphology at different time points after insertion into the skin.

After successfully preparing the microneedle patch, it was photographed using a digital camera, as shown in Figures 3a–c. The prepared GM-Clin-MN was a 2 cm × 2 cm square with a needle array of 20 × 20. The needle tips were sharp, intact and semi-transparent, which were connected well to the base. Measurements using ImageJ software revealed that the needle tips were approximately 500 ± 10 μm × 500 ± 10 μm × 800 ± 15 μm pyramidal shapes, with a spacing of 700 ± 8 μm between the tips (n = 3) as shown in Table 1. As shown in Figure 3d, the base layer of the microneedle was flexible and could be curled or folded freely. This suggested that the microneedle could be trimmed to accommodate various sizes. The red arrow in Figure 3e indicated that the microneedle can fit tightly on the joint surface and exhibited certain adhesive properties.

**TABLE 1:** Microneedle dimensional parameters (n = 3).

Parameter	Value (Mean ± SD)
Patch size	2 cm × 2 cm
Array configuration	20 × 20 needles
Needle height	800 ± 15 μm
Pyramid base	500 ± 10 μm × 500 ± 10 μm
Inter-needle spacing	700 ± 8 μm

SEM images of the microneedle were presented in Figures 3f,g. The base layer of the microneedle appeared very smooth, the needle tips were well-arranged, and the needle body surface was slightly rough but structurally intact. To visualize drug loading, a hydrophilic fluorescent dye, rhodamine 6G, was used as a simulated drug and observed under a fluorescence microscope. Figure 3h,i showed the red fluorescence emitted by the needle tips, indicating the successful loading of rhodamine 6G into the microneedles.

Figure 3j displayed the FTIR spectra of GM-Clin-MN, GM-MN, and Clin. The infrared spectrum of hydrochloride clindamycin showed characteristic peaks at 1,682 cm^−1^ (C=O), 1,080 cm^−1^ (C-O), 1,551 cm^−1^ (C=C), and 3,273 cm^−1^ (C-H). The infrared spectrum of GelMA showed characteristic peaks at 1,630 cm^−1^ (C=C), 1,533 cm^−1^ (C-N-H), and 3,273 cm^−1^ (C-H). FTIR spectroscopy analysis revealed that GM-Clin-MN exhibited enhanced peak intensity at 3,273 cm^−1^ (C-H), but no significant peak shifts or new characteristic peaks were observed.

Figure 3l showed the *in vitro* release curve of clindamycin hydrochloride. As shown in the graph, on the first day, the drug was released rapidly, with an accumulated release rate of (54.8 ± 2.1)%. Afterward, the drug was released slowly, and on day 10, the accumulated release rate reached (72.1 ± 1.5)%.

As shown in Figure 3m, under the universal testing machine, the microneedle underwent continuous deformation without any obvious inflection point. The pressure on the microneedle was positively correlated with the deformation of the needle tip. The mechanical strength of GM-Clin-MN was slightly higher than that of GM-MN. However, when the deformation of the needle tip reached 0.3 mm, both types of microneedles experienced a pressure greater than 0.50 N/Needle.

To assess skin penetration, the insertion ratio and insertion depth of the microneedle into the abdominal skin of SD rats were measured using the abdominal skin of SD rats. As shown in Figure 3n, after the microneedle loaded with rhodamine 6G was inserted into the skin for 1 min, the red dot on the skin surface represents the remaining rhodamine 6G inside the skin, indicating successful insertion of the microneedle into the skin. The insertion ratio was calculated to be 100% (n = 3). H&E staining of tissue slices in Figure 3o revealed that the prepared microneedles penetrated the skin to a depth of (658 ± 66) μm.

As shown in Figure 3p, after the microneedle was inserted into the abdominal skin of SD rats, the needle tip swelled, and the base of the needle widened over time, while the tip dissolved. The swelling ratio (S) of the microneedle was (185.4 ± 12.1)%. At 10 min, the separation between the needle tip and the base layer was observed, confirming the detachable characteristic of the prepared microneedles.

### 3.3 *In vitro* performance evaluation

**FIGURE 4:**
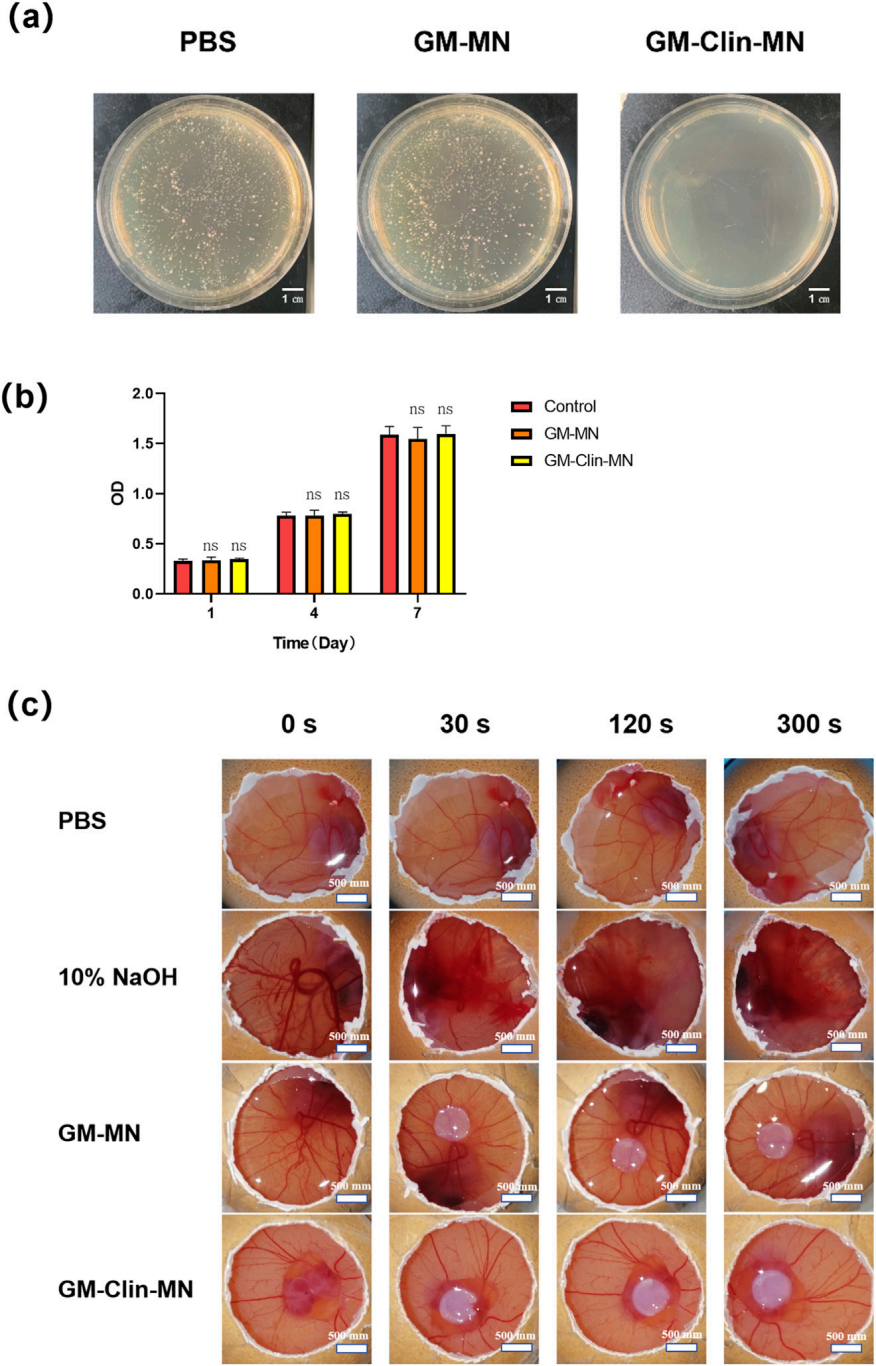
*In vitro* biological performance evaluation of GelMA. **(a)** Comparative images of *in vitro* antimicrobial experiment of microneedles against C. acnes. **(b)** Cell viability after culture for 1, 4 and 7 days. **(c)** Comparative images of HET-CAM irritation tests. ns: no significant differences, indicates *p* > 0.05 (n = 3).

The antimicrobial performance of the microneedles was validated by co-culturing with *C. acnes*. As shown in Figure 4a, no *C. acnes* colonies were observed on the culture dish of GM-Clin-MN, while a large number of individual *C*. acnes colonies were visible on the blank microneedles (GM-MN) and PBS culture dishes, with no significant difference between them. This indicates that GM-MN did not exhibit significant antimicrobial properties, while the clindamycin hydrochloride-loaded microneedles (GM-Clin-MN) showed excellent antimicrobial performance with a maximum inhibition rate of 100% (*n* = 3).

The viability of HSF seeded on the surface of the microneedles was detected using the CCK-8 assay on days 1, 4, and 7. The results, shown in Figure 4b, demonstrated that the number of HSF cells on the surfaces of the different materials gradually increased with prolonged culture time, but there was no significant difference among the groups (*p > 0.05*), indicating that both GM-MN and GM-Clin-MN had no apparent cytotoxicity and supported good cell growth.

The HET-CAM experiment results in Figure 4c; Table 2 showed that the positive control group treated with 10% NaOH exhibited bleeding and vascular dissolution within 30 s, which worsened with time. However, both GM-MN and GM-Clin-MN microneedles did not induce bleeding, clotting, or vascular dissolution on the CAM and presented an irritation score of 0, indicating their non-irritating nature. Combined with the above-mentioned CCK-8 cytotoxicity experiment results, it was verified that the microneedle patches prepared in this study possessed good adaptability and biocompatibility when applied to the skin.

**TABLE 2:** Classification of cumulative scores in the HET-CAM test (n = 6).

Groups	IS (RSD%)	Results
PBS	0 (0)	Non-irritant
10% NaOH	11.98 (1.06)	Extremely irritant
GM-MN	0 (0)	Non-irritant
GM-Clin-MN	0 (0)	Non-irritant

IS, irritation score; RSD%, relative standard deviation in percentage.

### 3.4 *In vivo* performance evaluation

**FIGURE 5:**
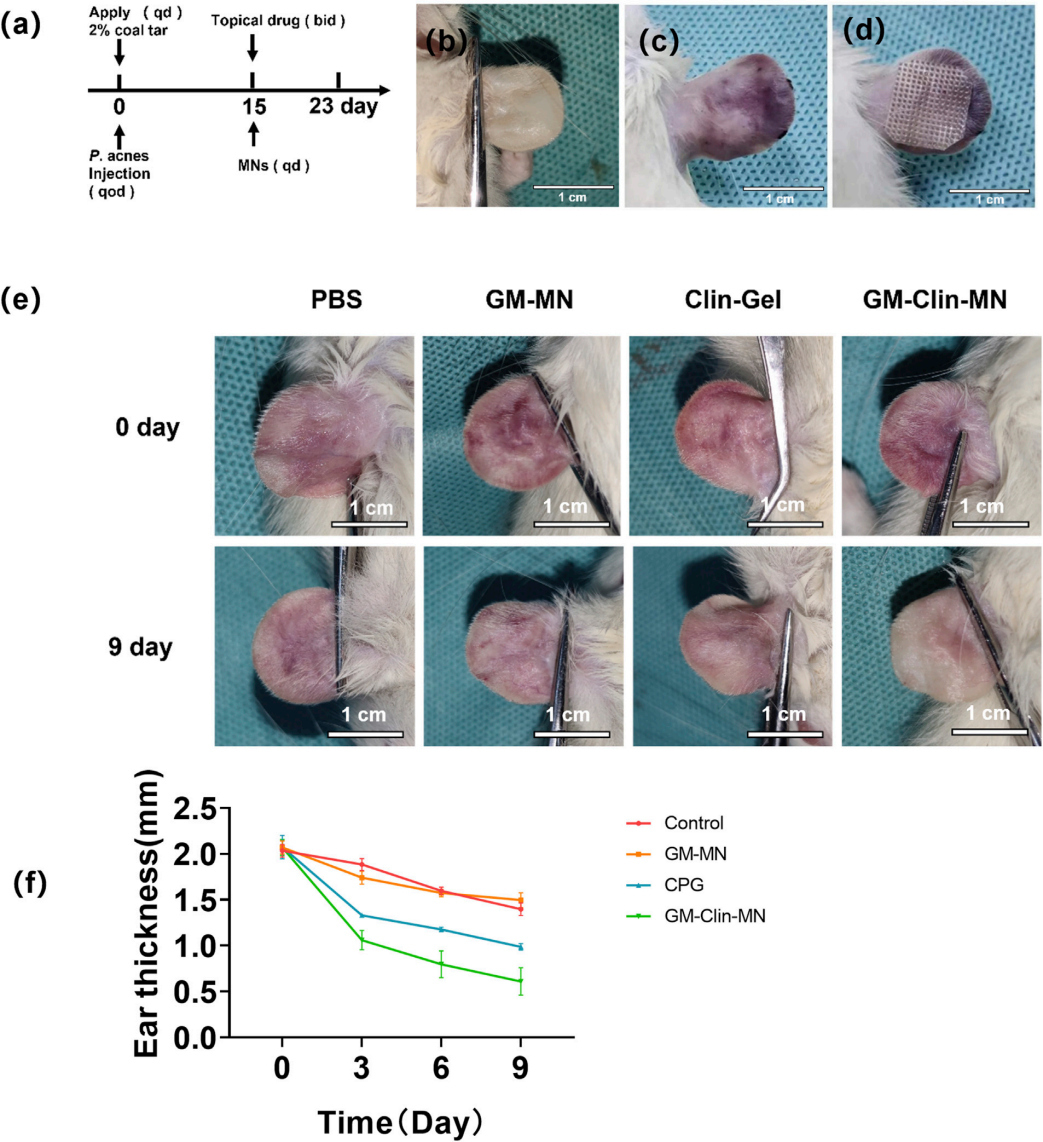
*In vivo* evaluation of GM-Clin-MN treatment for rat ear acne. **(a)** Time course diagram of acne animal treatment. Qd: once a day. Qod: every other day. Bid: twice a day. **(b)** Normal rat ear. **(c)** Rat ear after acne induction. **(d)** Illustration of microneedles applied to the rat ear for acne treatment. **(e)** Comparative image after acne treatment in rats. (PBS: negative control group. GM-MN: blank microneedle group). **(f)** Changes in rat ear thickness with treatment duration.

The modeling and treatment timeline for the rat ear acne model were shown in Figure 5a. Figure 5b illustrates the normal rat ear anatomy, while Figure 5c depicts the acne model morphology post-successful induction. On day 15, microneedle patches were applied to the skin lesions on the rat’s ear (Figure 5d) for a combined treatment period of 9 days. Figure 5e provided an overall morphological comparison of the lesions after treatment in each group. Figure 5f; Table 3 showed the change in ear thickness over the course of treatment. In the PBS group, there was no significant difference in the appearance of the skin lesions before and after treatment as observed by the naked eye. In the GM-MN group, after 9 days of treatment, the color of the skin lesions slightly lightened, and the swelling was slightly reduced, but there was no significant decrease in cysts and nodules. On day 3, the ear thickness was slightly thinner compared to the PBS control group, and the statistical analysis showed significance (p < 0.05). In the Clin-Gel group, after 9 days of treatment, the color of the skin lesions became lighter, the swelling reduced, and there was a partial decrease and dissipation of cysts and nodules. In the GM-Clin-MN group, after 9 days of treatment, the color of the skin lesions significantly lightened, mostly returning to the rat’s ear’s original color. Swelling noticeably reduced, and most of the cysts and nodules disappeared. On day 3, 6, and 9, the ear thickness significantly decreased compared to the PBS and Clin-Gel groups, and the statistical analysis showed significance (*p < 0.05*).

**TABLE 3:** Changes in rat ear thickness with treatment duration.

Groups	n	Time/d			
		0	3	6	9
PBS	10	2.03 ± 0.05	1.88 ± 0.05	1.59 ± 0.03	1.39 ± 0.06
GM-MN	10	2.07 ± 0.06	1.64 ± 0.06^*^	1.57 ± 0.03	1.50 ± 0.07
CLin-Gel	10	2.07 ± 0.10	1.33 ± 0.06	1.17 ± 0.07	0.98 ± 0.03
GM-Clin-MN	10	2.07 ± 0.07	1.06 ± 0.09^*#^	0.80 ± 0.12^*#^	0.61 ± 0.12^*#^

Significance markers: *P < 0.05 vs. PBS; #*P* < 0.05 vs. Clin-Gel.

**FIGURE 6:**
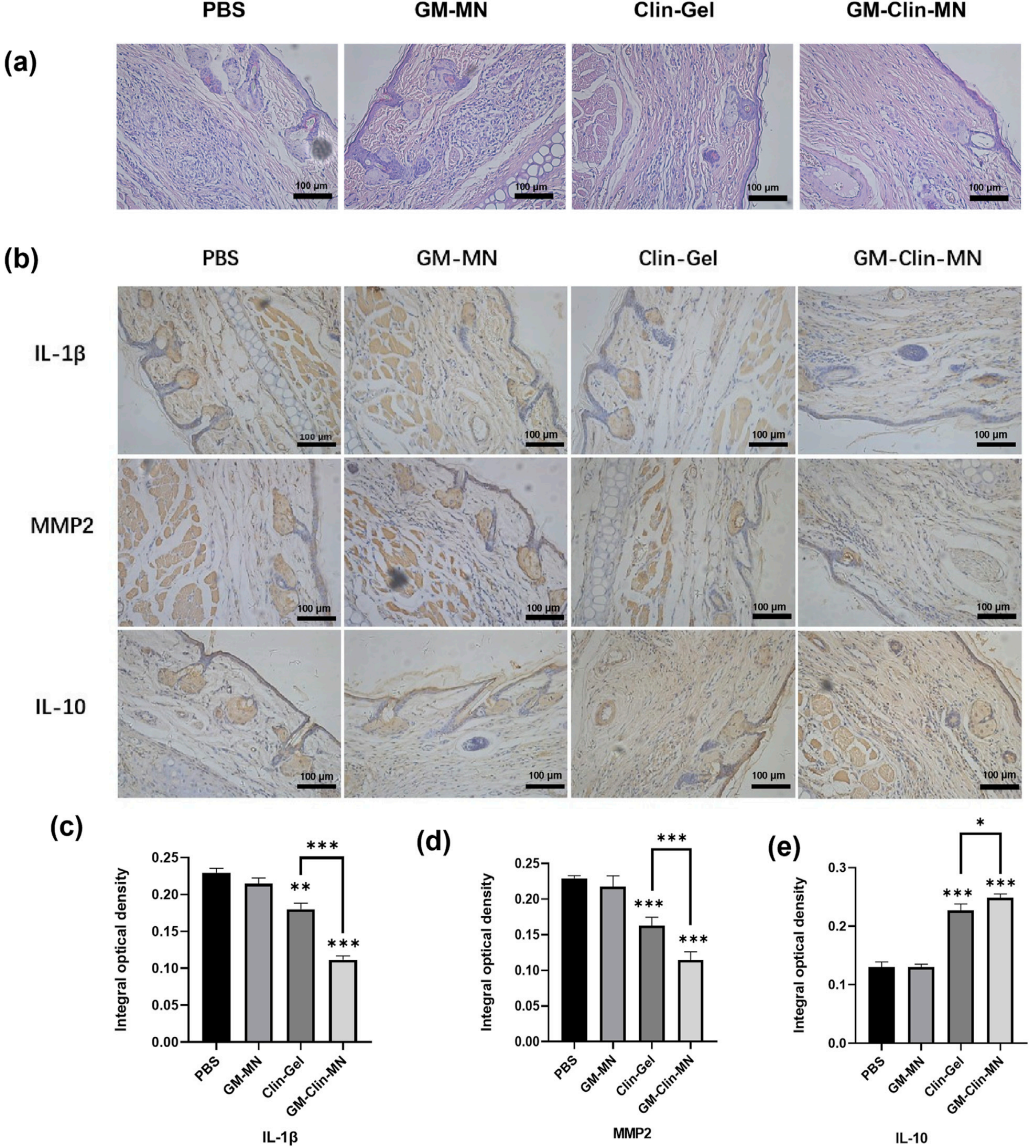
**(a)** Histological images of treated acne rat model tissue stained with HE (×200). **(b)** Protein expression levels of IL-1β, MMP-2, and IL-10 in rat ear tissue among different groups. **(c)** Comparative graph of IL-1β protein expression IOD (Integrated Optical Density) values. **(d)** Comparative graph of MMP-2 protein expression IOD values. **(e)** Comparative graph of IL-10 protein expression IOD values. (* indicates *p* < 0.05, ** indicates *p* < 0.01, *** indicates *p* < 0.001; n = 3).

The tissues post-treatment were stained with Hematoxylin and Eosin (HE), and the results were depicted in Figure 6a; Table 4. In comparison to normal rat ear tissue, both the PBS group and GM-MN group displayed significant structural damage to the ear tissue. The epidermal spinous layer thickened, hair follicles dilated with an accumulation of keratinocytes, and collagen fibers in the dermis thickened and exhibited disorganized arrangement. Capillaries in the dermal layer were dilated, and there was noticeable infiltration of inflammatory cells. Compared to the PBS and GM-MN groups, the Clin-Gel group demonstrated a restoration of normal ear structure. The epidermal spinous layer became thinner, infiltration of inflammatory cells in the dermal layer decreased, and dilation of capillaries was reduced. However, some hair follicle openings still showed slight dilation. In contrast to the previous three groups, the GM-Clin-MN group exhibited clear boundaries between ear tissue layers. Contents in the hair follicles were smaller and less abundant, and sebaceous glands had a normal volume. Collagen fibers in the dermal layer were well-organized, inflammatory cell infiltration was minimal, and capillary dilation was not apparent.

**TABLE 4:** Histopathological grading of rat ear skin lesions.

Groups	PBS			GM-MN			CLin-Gel			GM-Clin-MN		
	A	B	C	A	B	C	A	B	C	A	B	C
Abnormal changes	+++	+++	+++	++	+++	+++	++	++	++	+	+	+
Hyperkeratosis	3	3	2	3	2	3	2	1	2	1	2	0
Inflammatory cells infiltration	3	2	3	2	3	3	2	3	2	0	1	1
Thick epidermis (hypertrophy)	2	2	2	2	3	3	2	2	1	2	1	2
Dermal congestion	3	3	2	2	3	2	3	2	2	0	0	0
Hair follicles dilation	2	3	3	3	2	2	1	1	3	1	0	1

Grade of histopathological findings: (No abnormality), + (Mild), ++ (Moderate), +++ (Severe).

Immunohistochemistry was performed to detect the expression of IL-1β, MMP2, and IL-10 in rat ear tissues, and the results are shown in Figure 6b. The brownish-yellow particles represent positive expression. In terms of the expression of the pro-inflammatory factors IL-1β and MMP2, compared to the PBS group, both the Clin-Gel and GM-Clin-MN groups showed a significant reduction in the area and intensity of positive staining, while the GM-MN group showed no significant change. Compared to the Clin-Gel group, the GM-Clin-MN group exhibited a significant decrease in the area and intensity of positive staining. For the anti-inflammatory factor IL-10, both the Clin-Gel and GM-Clin-MN groups showed a significant increase in the area and intensity of positive staining, while the GM-MN group showed no significant change. Compared to the Clin-Gel group, the GM-Clin-MN group exhibited a significant increase in the area of positive staining and a slight deepening of color.

Using ImageJ software, we calculated and analyzed the integrated optical density (IOD) values of MMP-2, IL-10, and IL-1β protein expression, as shown in Figures 6c–e. Compared to the PBS group, the Clin-Gel group exhibited a modest reduction in the expression of the pro-inflammatory factor IL-1β (P = 0.0011), while the GM-Clin-MN group showed a significant decrease in IL-1β expression (P = 0.0001), with a 90.90% reduction in P-value compared to the Clin-Gel group. Similarly, compared to the PBS group, the Clin-Gel group showed a slight decrease in the expression of the pro-inflammatory factor MMP2 (P = 0.0007), whereas the GM-Clin-MN group demonstrated a significant reduction in MMP2 expression (P = 0.0001), with an 85.71% reduction in P-value compared to the Clin-Gel group. In comparison to the PBS group, the Clin-Gel group exhibited a slight increase in the expression of the anti-inflammatory factor IL-10 (P = 0.0003), while the GM-Clin-MN group showed a significant increase in IL-10 expression (P = 0.0001), with a 66.67% reduction in P-value compared to the Clin-Gel group.

## 4 Discussions

Clindamycin, as one of the most widely used antibiotics for treating acne, exerts direct bactericidal effects on *C. acnes* within the sebaceous follicles and also possesses anti-inflammatory properties. However, being a hydrophilic drug, it faces challenges in transdermal penetration due to the hydrophobic nature of the stratum corneum when applied topically. Traditional local formulations of clindamycin, such as gels and creams, may lead to certain side effects, including skin irritation, erythema, dryness, peeling, and burning (Armillei et al., 2024). Additionally, clindamycin hydrochloride has difficulty penetrating the stratum corneum of the skin, resulting in low bioavailability of these formulations, typically around 5%–8% (Saeedi et al., 2024). Some researchers have explored nanoparticle carriers to enhance transdermal delivery of clindamycin (Chakraborty et al., 2022), however, few studies have systematically investigated the combination of clindamycin hydrochloride with hydrogel microneedles for acne treatment. Currently, hydrogel microneedles have attracted great attention in transdermal drug delivery (Filho et al., 2023). Clindamycin hydrochloride-loaded GelMA microneedles (GM-Clin-MN) in this study achieved a tip drug loading of 0.49 ± 0.025 μg/needle, with rapid release (54.8% ± 2.1% on Day 1) and sustained kinetics (72.1% ± 1.5% by Day 10). The needles exhibited mechanical strength >0.50 N/needle (deformation limit: 0.3 mm), enabling stratum corneum penetration, and post-insertion swelling of 185.4% ± 12.1%. Tips detached fully from the base within 10 min. GM-Clin-MN showed no cytotoxicity or skin irritation and 100% inhibition against *C. acnes.* In rat models, it significantly reduced IL-1β and MMP-2 expression (*P < 0.05*), elevated IL-10 (*P < 0.05*), and suppressed C. acnes-induced inflammation, demonstrating anti-scarring potential via MMP-2 downregulation.

In the pathogenesis of acne, the colonization of *C. acnes* and the inflammatory response play a key role in the occurrence and development of inflammatory acne. In this process, the excessive secretion of sebum by sebaceous glands leads to the blockage of the pilosebaceous unit, creating an excellent environment for the proliferation of anaerobic *C. acnes*. These bacteria release various enzymes that can break down sebum into free fatty acids. This not only exacerbates the hyperproliferation and hyperkeratinization of the pilosebaceous duct but also stimulates the follicular wall to express pro-inflammatory cytokines (Lesiak et al., 2024). Severe inflammatory acne lesions often present with erythema, pigmentation, and permanent scars, which have a significant negative impact on the patient’s life and psyche. Currently, the main topical antibiotics used in the treatment of acne are erythromycin and clindamycin. They primarily work by directly inhibiting the antibacterial activity of *C. acnes* and indirectly reducing inflammation (Niedźwiedzka et al., 2024). Among these two drugs, clindamycin is the preferred option because erythromycin tends to develop resistance more easily, leading to a significant decrease in its effectiveness over time (Armillei et al., 2024). However, clindamycin is a hydrophilic drug, and due to the hydrophobic nature of the stratum corneum, it is unable to effectively penetrate the skin barrier when applied topically. To address this issue, clindamycin hydrochloride was incorporated into GM-MNs to enhance transdermal efficiency. To verify the effective loading of hydrophilic substances into GM-MNs, we used the hydrophilic fluorescent dye rhodamine 6G as a model drug and visualized it under a fluorescence microscope. The experimental results demonstrate that GM-MNs can successfully load rhodamine 6G, indicating that these microneedles can effectively load hydrophilic antibiotic clindamycin hydrochloride. Compared to GM-MNs, the peaks attributed to clindamycin were observed in GM-Clin-MNs, indicating clindamycin was successfully loaded into the microneedles. Moreover, no additional new characteristic peaks were observed in GM-Clin-MNs, suggesting no new compounds were formed when clindamycin hydrochloride was mixed with GelMA. By ensuring the stability of the drug, they can improve transdermal efficiency and enhance its bioavailability.

The half-life of clindamycin hydrochloride is approximately 3 h. In clinical practice, the frequency of intravenous or intramuscular administration of clindamycin hydrochloride injection is four times daily with relatively short intervals (Radu et al., 2024). In the treatment of common acne, it is typically necessary to take oral antibiotics continuously for 6–8 weeks, which is a relatively long duration. The local transdermal effect of topical clindamycin hydrochloride gel is not satisfactory (Syder et al., 2025). Therefore, it is necessary to find a suitable biodegradable carrier material and modify it to achieve sustained release performance. This study developed a sustained and controlled release clindamycin hydrochloride-loaded microneedle patch based on a gelatin methacryloyl (GelMA) hydrogel for acne treatment, which exhibited a biphasic release profile: an initial burst release phase (54.8% ± 2.1% cumulative release within 24 h) followed by a sustained release phase (72.1% ± 1.5% cumulative release over 10 days). The burst release was primarily attributed to the rapid diffusion of surface-adsorbed drug molecules, pore expansion induced by hydrogel swelling during hydration, and partial degradation of the superficial matrix, while the sustained release phase relied on the stability of the GelMA crosslinked network and gradual drug release from deeper layers. Experimental results demonstrated that this biphasic pattern aligned well with therapeutic requirements for acne—the burst phase rapidly suppressed *C*. *acnes* proliferation and alleviated acute inflammation through high drug concentrations, whereas the sustained phase maintained sub-therapeutic drug levels to prevent bacterial regrowth and reduce resistance risks. This technology not only addresses the limitations of frequent administration in conventional topical formulations but also provides an innovative delivery strategy integrating rapid response and long-term efficacy for chronic dermatological conditions. Future studies should focus on *in vivo* studies to validate its local safety and long-term efficacy. Another study loaded clindamycin hydrochloride onto carboxymethyl chitosan nanoparticles cross-linked with Ca^2+^ ions (CMCS-Ca^2+^) to achieve sustained release of hydrophilic antibiotics. The drug release rate was (69.88 ± 2.03)% at 60 min and gradually increased to (94.99 ± 4.70)% at 24 h (Chaiwarit et al., 2022). In comparison, GM-Clin-MN exhibited a longer sustained release duration, making it promising for diseases that require long-term maintenance of clindamycin hydrochloride therapeutic effects.

During the manufacturing process, drugs can be incorporated into the polymer structure or loaded into a separate reservoir and attached to the microneedles. After insertion into the skin, such drug-loaded microneedles can be used for transdermal drug delivery (Nguyen et al., 2025). Hydrogel microneedles also overcome some limitations of traditional silicon or metal microneedles. They have higher drug-loading capacity and adjustable drug release rates, which are often related to the polymer crosslinking density (Omidian and Dey Chowdhury, 2025). The GM-Clin-MN prepared in this experiment is a 2 cm × 2 cm square with a needle tip array of 20 × 20. The needle tips have a pyramid shape with dimensions of approximately 500 ± 10 μm × 500 ± 10 μm × 800 ± 15 μm, and the spacing between needle tips is 700 ± 8 μm (n = 3).

In this study, the molecular weight of PVA used for the microneedle substrate layer was 89,000–98,000 Da. This molecular weight was selected as it balances the flexibility and skin adhesion of the microneedle substrate layer while maintaining rapid degradation characteristics—lower molecular weight PVA lacks sufficient mechanical strength, while higher molecular weight PVA tends to become brittle (Starlin Chellathurai et al., 2023). The microneedle base layer is flexible and can be easily rolled up or folded. It can be cut to any size depending on the requirements. Therefore, when using these microneedles for acne treatment, they can be further cut according to the size and morphology of the skin lesions, meeting individual needs. Acne lesions have uneven surfaces, which differ from the smooth surface of healthy skin. To simulate the uneven skin surface, the microneedles are applied to the joint surface of the finger. The microneedles can tightly adhere to the joint surface, demonstrating certain adhesive properties. These characteristics make GM-Clin-MN a potential new treatment for acne. Scanning electron microscopy images of GM-Clin-MN show a very smooth base layer, and the needle tips are full and intact, but the surface of the microneedle shaft is slightly rough, possibly due to the degree of crosslinking of GelMA. This phenomenon is similar to the microneedles prepared by Zhou et al., and the higher roughness also reflects a higher degree of crosslinking in hydrogel microneedles (Zhou et al., 2023).

FTIR analysis showed overlapping C-H stretching bands at 3,273 cm^−1^ between clindamycin hydrochloride and GelMA, with increased peak intensity reflecting additive contributions rather than chemical interactions. No new peaks emerged in GM-Clin-MN compared to GM-MN, confirming the absence of covalent bonding. The lack of significant peak shifts suggests physical entrapment or weak hydrogen bonding within the GelMA matrix, which insufficiently altered vibrational modes. While FTIR effectively identifies covalent modifications, it has limited sensitivity for weak non-covalent interactions.

Factors influencing the mechanical strength of polymer microneedles include the material composition, shape, aspect ratio, and environmental humidity of the microneedles. In this study, we used pyramid-shaped microneedles because, under the same base width or diameter, pyramid-shaped microneedles have greater mechanical strength compared to conical microneedles (Choo et al., 2022). The viscoelasticity of the skin reduces the penetrating force of microneedles, so microneedles must have sufficient mechanical strength to penetrate the skin. Studies have shown that the insertion force required for microneedles to penetrate the skin barrier is approximately 0.098 N/needle (Al-Badry et al., 2023). When the needle tip deformation of GM-Clin-MN reached 0.3 mm, the pressure exerted on the needle tip exceeded 0.50 N/needle, proving that the microneedles have superior mechanical strength to effectively penetrate the stratum corneum of the skin and promote transdermal drug delivery. In this study, we used the shaved abdominal skin of SD rats to measure the insertion ratio and insertion depth of microneedles *in vitro*. Results from microneedles loaded with rhodamine 6G and inserted into the skin for 1 min showed successful penetration of the microneedles, with a calculated insertion ratio of 100%. H&E staining of the inserted skin tissue slices further confirmed this result. The depth of microneedle insertion into the skin was (658 ± 66) μm, which is less than the actual height of the microneedles (approximately 800 μm), possibly due to the elasticity of the skin. However, the depth of microneedle insertion into the skin is sufficient to penetrate the stratum corneum (approximately 10–20 μm) and reach the dermis, facilitating effective drug delivery in the dermis (Zhao et al., 2023). After inserting GM-Clin-MN into the abdominal skin of SD rats, over time, the needle tip of the microneedle swells, the base of the needle tip widens, and the microneedle tip separates from the base. The tip then acts on the dermis layer of the skin. This phenomenon is consistent with the research results of Ge et al. (2024). Microneedles prepared using cross-linked biopolymer materials can achieve swelling rather than dissolution of the microneedles in the subcutaneous tissue. Increasing the weight percentage of the crosslinker, such as PEG, results in a higher crosslinking density, thereby reducing the expansion capability of the microneedles. This tunable expansion ability is associated with the drug release rate. By controlling the expansion ability, the drug release rate can be manipulated to the desired level by altering the degree of crosslinking. The dynamics of drug delivery depend on the size of the hydrogel pores and the size of the solute being delivered, as the hydrogel matrix acts as a permeable barrier. The mesh size of the hydrogel indicates the size and spacing of the pores. If the solute to be delivered is larger than the mesh size, the drug cannot be delivered. However, upon swelling, the mesh size increases, allowing the drug to be released (Aroche et al., 2024; Omidian and Dey Chowdhury, 2025). Therefore, faster swelling results in a more rapid drug release (Qi et al., 2024). Within 10 min, the separation of the needle tip from the base layer was observed, confirming the detachable characteristic of the prepared microneedles and improving the convenience of microneedle use.

The human skin provides a physiological barrier to protect internal organs from temperature changes, mechanical impacts, microorganisms, and various chemicals (Liu et al., 2025). When the skin is exposed to various chemicals and biological agents, it can trigger various skin diseases such as inflammation, allergies, and even cancer (Yin et al., 2025). Therefore, it is necessary to assess the toxicity and skin irritation response of materials and drugs to the skin when preparing topical formulations. The cytotoxicity evaluation served two primary purposes: (1) to verify the biocompatibility of the microneedle matrix materials (GelMA/PVA), and (2) to assess potential adverse effects of clindamycin-loaded formulations on normal skin cells (HSF). These studies established a critical safety foundation for subsequent *in vivo* applications. In this experiment, human skin fibroblasts were co-cultured with microneedles, and the cell viability was assessed using the CCK-8 assay. The results showed that GM-MN and GM-Clin-MN did not exhibit significant cytotoxicity. Zhang et al. suggested that an increase in hydrogel concentration promotes cell proliferation because the internal structure of the hydrogel becomes more compact, providing better adhesive contact points for the cells (Zhang et al., 2020). In this study, GelMA did not show a significant effect on promoting cell proliferation, which may be related to the concentration of GelMA and the duration of photocrosslinking. However, it exhibited good biocompatibility with human skin fibroblasts (HSF) and can safely interact with the dermal layer of the skin. The HET-CAM assay has been widely used to assess the irritancy of topical formulations and avoids the need for testing on live animals with a similar purpose (Shetty et al., 2025). Similar to the placenta in mammals, the extraembryonic structures (allantois, chorion, yolk sac, and amnion) develop within the eggshell in birds. The fusion of the allantois and chorion forms the chorioallantoic membrane. A study developed chitosan and hyaluronic acid nanoparticles to encapsulate clindamycin and improve its transdermal efficiency, and the HET-CAM assay was conducted to evaluate the safety of this formulation (Tolentino et al., 2021). Therefore, in this experiment, this method was chosen to assess the irritancy of GM-Clin-MN to the skin, and changes in toxicity indicators of the CAM, such as bleeding, clotting, and vascular lysis. The results showed no bleeding, clotting, or vascular lysis in the CAM of the GM-MN and GM-Clin-MN groups, indicating their non-irritating nature. Combined with the aforementioned CCK-8 cytotoxicity assay results, it confirms that the microneedle patch prepared in this study exhibits good adaptability and biocompatibility when applied to the skin.

The pathogenesis of acne is complex, with four main contributing factors: abnormal androgen levels, excessive sebum production, abnormal keratinization of follicular cells, and inflammatory reactions (primarily induced by *C. acnes*). Due to the coexistence of diverse acne lesions such as comedones, papules, pustules, and nodules, neither chemical irritation alone nor subcutaneous injection of *C. acnes* suspension can fully replicate the morphological features of acne. Therefore, based on previous literature and the Kligman method (Fu et al., 2024), this study established a rat ear acne model by daily topical application of 2% coal tar combined with alternate-day subcutaneous injection of *C. acnes*.

The application of 2% coal tar at the opening of the rat ear duct simulates skin barrier disruption and excessive sebum secretion, leading to follicular keratinization and creating an anaerobic environment conducive to *C. acnes* proliferation. Meanwhile, subcutaneous injection of *C. acnes* suspension mimics bacterial overgrowth-induced immune responses and inflammation. This combined approach better replicates the pathophysiological process of acne.

After successful modeling, GM-MN and GM-Clin-MN patches were applied to the rat ear lesions. PBS was used as a negative control, and clinical-grade clindamycin hydrochloride gel was used as a positive control. The combined treatment lasted for 9 days. The macroscopic analysis of the lesion appearance showed that in the GM-MN group on day 3, the thickness of the rat ear was slightly thinner compared to the PBS control group, and the statistical analysis showed significance (*p < 0.05*). This phenomenon may be attributed to the adsorption of some pus due to the swelling effect of the cross-linked hydrogel after GM-MN attachment to the acne lesion. This result is consistent with the study by Zhang et al. (Zhang et al., 2018). After 9 days of treatment in the GM-Clin-MN group, the color of the lesions became noticeably lighter, most of them returned to the original color of the rat ear, swelling was significantly reduced, and most cysts and nodules disappeared. On days 3, 6, and 9, the ear thickness in the GM-Clin-MN group was significantly reduced compared to the PBS group and Clin-Gel, and the statistical analysis showed significance (*p < 0.05*). Therefore, it can be concluded that GM-Clin-MN has excellent therapeutic effects on the rat model of acne. In addition to the antibacterial effect of clindamycin hydrochloride, the absorbency of the microneedle patch may also play a role in the absorption of pus.

After treatment, the tissues were subjected to H&E staining. Compared to the other three groups, the GM-Clin-MN group exhibited clear boundaries between ear tissue layers. Contents in the hair follicles were smaller and less abundant, and sebaceous glands had a normal volume. Collagen fibers in the dermal layer were well-organized, inflammatory cell infiltration was minimal, and capillary dilation was not apparent. Therefore, from a histological perspective, GM-Clin-MN demonstrates excellent therapeutic effects on acne compared to Clin-Gel, which is consistent with the macroscopic observations of the rat ear acne model after treatment.

During the acne inflammation process, both cellular immunity and humoral immunity are involved as well as various cytokines. Acne *C. acnes* stimulates Toll-like receptors (TLRs) on keratinocytes to produce various pro-inflammatory cytokines such as IL-1β, IL-6, IL-8, and IL-12, which are released into the extracellular environment and participate in follicular inflammation (Feng et al., 2024). With the abundant colonization of *C. acnes* in sebaceous follicles, it can also stimulate the expression of matrix metalloproteinases (MMPs), especially MMP-2. These enzymes are associated with the inflammatory response of acne and play an important role in extracellular matrix degradation and scar formation after acne (Xu et al., 2024). IL-10 is mainly produced by Th2 cells and is a broad-spectrum anti-inflammatory factor. Lesiak et al. evaluated the levels of IL-10 and other cytokines in the serum of acne patients and found that the serum IL-10 level was negatively correlated with disease severity, suggesting that IL-10 has anti-inflammatory effects in the inflammatory response of acne (Lesiak et al., 2024). Another study has shown that in addition to inhibiting the synthesis of IL-1β and other pro-inflammatory factors, IL-10 can also promote the production of tissue inhibitors of matrix metalloproteinases (TIMPs), thereby inhibiting the expression of MMPs and reducing scar formation in acne (Murakami and Shigeki, 2024). Therefore, in order to understand the expression of inflammation in the acne model, we selected the pro-inflammatory cytokine IL-1β, MMP-2, and the anti-inflammatory cytokine IL-10 as the detection indicators in this experiment to observe their expression levels in the acne inflammation model. The results show that Clin-Gel and GM-Clin-MN can reduce the expression of pro-inflammatory factors IL-1β and MMP-2 in acne lesions, increase the expression of anti-inflammatory factor IL-10, and GM-Clin-MN has a significantly better effect (*p < 0.05*), indicating that GM-Clin-MN has a significant inhibitory effect on the acne inflammation induced by acne *C. acnes*. The observed MMP-2 downregulation (*p < 0.05*) could contribute to mitigating acne-related scarring, consistent with its established role in collagen remodeling (Bhavsar et al., 2014). However, definitive evidence of scar reduction requires longitudinal histological validation, including quantitative analysis of collagen fiber density and orientation (e.g., via polarized light microscopy). In this study, a rat ear acne model was established using a combined approach of daily topical application of 2% coal tar and alternate-day subcutaneous injection of *C. acnes*. However, this model still exhibits limitations: the sebaceous gland density in rat ear skin is significantly lower than that in human facial skin, and coal tar induces acute inflammation rather than mimicking the spontaneous comedone formation observed in humans. Additionally, the model does not incorporate the hormonal fluctuation mechanisms associated with puberty. Therefore, more effective approaches for constructing acne animal models warrant further exploration. The clindamycin sustained-release microneedle patch (GM-Clin-MN) prepared in this experiment is expected to be further evaluated for its efficacy and safety through clinical trials.

## 5 Conclusion

This study provides a simple and effective strategy for treating acne infection by combining clindamycin hydrochloride with GelMA microneedles. Loading clindamycin hydrochloride into GelMA microneedles ensures that it can facilitate painless delivery into the microchannels on the skin surface. The microneedles exhibit high mechanical performance, allowing for sustained drug delivery for more than 10 days. Additionally, compared to clindamycin hydrochloride gel used in clinical applications, the microneedles accelerated the transdermal delivery efficiency of clindamycin hydrochloride and enhanced the therapeutic effect *in vivo*. This study represents the systematic investigation of the combination of clindamycin hydrochloride with GelMA microneedles for transdermal delivery in the treatment of acne, providing guidance for the design of new and effective topical therapeutic approaches. Despite these advances, limitations remain: the current formulation focuses on clindamycin alone, and degradation kinetics in sebum-rich environments require further optimization. Future studies will explore combination therapies (e.g., clindamycin + epigallocatechin gallate) and pilot-scale GMP production. This work not only advances transdermal acne management but also provides a template for engineering polymer-based microneedles against other cutaneous disorders.

## Data Availability

The raw data supporting the conclusions of this article will be made available by the authors, without undue reservation.

## Abbreviations

BHI, Brian heart infusion; Clin-Gel, Clindamycin hydrochloride gel; *C. acnes*, Cutibacterium acnes; DMSO, Dimethyl sulfoxide; FBS, Fetal bovine serum; FT-IR, Fourier transform infrared spectoscopy; GM-CLin-MN, Gelatin methylacryloy clindamycinl microneedle; GelMA, Gelatin methylacryloyl; GM-MN, Gelatin methylacryloy microneedle; HET-CAM, Hen’s egg test on the chorioallantoic membrane; HSF, Human skin fibroblasts; LAP, Llithium phenyl-2,4,6-trimethylbenzoylphosphinate; NMR, Nuclear magnetic resonance; PBS, Phosphate buffered saline; PDMS, Polydimethylsiloxane; PVA, Polyvinyl alcohol; SEM, Scanning electron microscope; TDDS, Transdermal drug delivery systems.
